# Anatomical predisposition and neurological vulnerability to brachial plexus birth injury: a contemporary narrative review

**DOI:** 10.1177/17531934261443088

**Published:** 2026-05-04

**Authors:** Hugo OH Beaumont, Akira Wiberg, Hazel Brown, Tom J Quick

**Affiliations:** 1 Musculoskeletal Research Unit, Medical School, University of Bristol, UK; 2 Nuffield Department of Orthopaedics, Rheumatology, and Musculoskeletal Sciences, University of Oxford, UK; 3 Southwest Brachial Plexus Team (SWePT), North Bristol NHS Trust, UK; 4 Centre for Nerve Engineering, University College London, UK

**Keywords:** Anatomy, birth injury, brachial plexus, Erb’s palsy, predisposition, vulnerability

## Abstract

Erb’s palsy, or brachial plexus birth injury (BPBI), is an injury sustained at birth to a vulnerable nervous system which even with the best care leaves many children with permanent impairments, growth retardation and pain. Established risk factors include maternal diabetes, increased foetal weight, shoulder dystocia and instrumental delivery. Yet these variables do not reliably predict which individual children will sustain injury. We propose an additional, largely neglected factor: developmental anatomical predisposition of the neonatal brachial plexus, with anomalies similar to those implicated in neurogenic thoracic outlet syndrome (nTOS) later in life. These include cervical ribs, aberrant scalene attachments and variant plexus branching – each capable of narrowing the supraclavicular corridor and concentrating mechanical strain during delivery. Such anatomical variants may lower the injury threshold and confer a ‘neurologic vulnerability’ when exposed to the forces of delivery. This may help explain why some infants sustain injury under normal obstetric conditions while others do not. We further propose that neonatal BPBI and adolescent nTOS represent distinct phenotypic expressions of a shared anatomical vulnerability, with a patterned molecular basis involving *Hox* gene regulation of axial skeletal patterning during embryogenesis. These insights carry direct clinical implications: in neonates undergoing brachial plexus exploration, recognition and decompression of anatomical compression may complement or replace standard nerve grafting strategies. We outline a research agenda including anatomical phenotyping, familial screening, biomarkers and prospective cohort studies to quantify this predisposition and integrate it into peripartum decision-making alongside conventional risk factors.

**Level of Evidence:** Level V

## Introduction

Brachial plexus birth injury (BPBI) presents at a rate of 0.4–4.6 per 1000 births worldwide and can have a significant negative impact on a child’s health-related quality of life (Akel et al., 2013; Dardas and Shah, 2021; Malessy and Pondaag, 2009). Despite modern obstetric care, outcomes for individuals and families remain suboptimal (Dorich et al., 2024; Yau et al., 2018). Identifying the risk factors for this injury and preventing injury remain ongoing challenges. Classical teaching emphasizes excessive traction on the presenting part during delivery complicated by shoulder dystocia as the principal aetiology (Dardas and Shah, 2021; Iffy, 2014). However, shoulder dystocia is relatively common compared with the incidence of BPBI, and plexus injury is also documented in deliveries without recognized dystocia or even after Caesarean section (Mollberg et al., 2005). These inconsistencies imply that the magnitude of obstetric force alone cannot fully account for the risk or severity of injury.

Considerable efforts have been made to reduce the forces applied to the head and neck of children during deliveries complicated by shoulder dystocia; however, many children still live with the lifelong impacts of BPBI (Blom et al., 2022). Surgical intervention and rehabilitation have a role, but the results are variable and often remain unsatisfactory to those living with BBPI and their families. Prevention of injury must be the responsibility of all working in this field.

Classical risk models for BPBI have focussed on maternal diabetes, estimated foetal weight, shoulder dystocia and the use of instruments (Mollberg et al., 2005). These factors are clearly contributory, yet they explain surprisingly little at the level of an individual birth. Many babies with one or more of these features never sustain a plexus injury while others are injured in deliveries that appear routine (Mollberg et al., 2005). The pattern and severity of lesions also vary widely, from transient upper-trunk weakness to pre-ganglionic avulsion, suggesting that the same external forces meet different internal thresholds. Taken together, these observations indicate that current explanations do not fully account for the observed variation. In adult brachial plexus injury, anatomic variation is recognized as feasibly being a contributing factor to both acute injury and in chronic compressive conditions of the brachial plexus (neurologic thoracic outlet syndrome, nTOS) (Benes et al., 2021; Patel and Smith, 2023).

The concept of developmental anatomical predisposition offers a possible explanatory model. Variants that narrow or alter the thoracic outlet and supraclavicular plexus corridor may focus or increase local strain during traction, lowering the threshold at which injury occurs. These same variations are familiar to peripheral nerve and thoracic outlet surgeons, as they contribute to the development of nTOS. Unlike obstetric forces, these anatomical features exist from early foetal development, creating the environment upon which delivery forces act.

This review examines the evidence for developmental predisposition in BPBI and considers how this framework could influence prevention of injury, clinical assessment and operative management.

## Embryology of the brachial plexus

**Figure 1.**
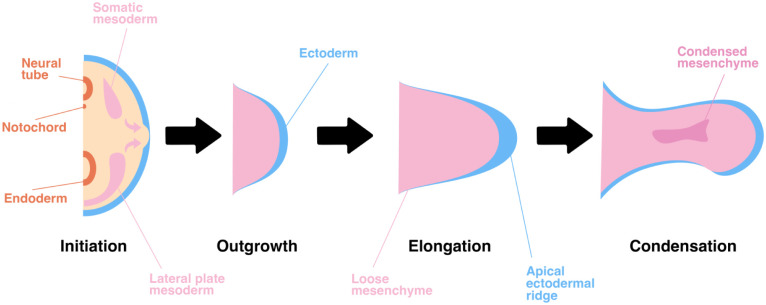
Stages of early limb bud development: initiation of limb bud growth from somatic and lateral plate mesoderm, outgrowth, elongation and condensation in which zones are formed which will eventually form musculoskeletal elements of the limb. Adapted from Al-Qattan et al. (2009) and Sermeus et al. (2022).

During early human development, the brachial plexus arises from the anterior (ventral) primary rami of spinal nerves C5 to T1 (Moore and Dalley, 2018). In parallel, around the fourth to fifth week of gestation, the embryo forms the upper-limb buds from the lateral plate mesoderm, which begin as ‘paddle-shaped’ mesenchymal outgrowths covered by ectoderm (Figure 1) (McQueen and Towers, 2020; Vargesson and Tickle, 2025).

As the limb bud enlarges, motor axons from the ventral roots extend into the mesenchymal core of the bud; soon after, sensory fibres from neural crest-derived dorsal root ganglia follow, resulting in a primitive nerve network supplying the developing upper limb. Simultaneously, mesodermal somites and lateral-plate mesoderm differentiate to form the skeletal, muscular, vascular and connective-tissue structures of the shoulder girdle and neck, including the future ribs, clavicle region, prevertebral musculature such as the scalenes and the subclavian and axillary vessels (Miller and Detwiler, 1936).

Mesodermal condensations corresponding to the fifth to seventh cervical ribs initially form during embryologic development and then regress to end up as transverse processes owing to their interaction with the brachial plexus nerve roots. The formation of a cervical rib is an expression of an error in this neurectoderm–mesoderm signalling, probably involving *Hox* gene expression (Galis, 1999; McNally et al., 1990). The standard patterns of brachial plexus anatomy are set by dynamic gene regulation networks during embryogenesis and the interaction between nerve root outgrowth and the simultaneous development of skeletal, muscular, vascular and connective tissues (Burke et al., 1995; Horan et al., 1995; Zuniga and Zeller, 2020). Variation in the development of any of these anatomical structures (for instance, rib formation, somite segmentation, or vessel position) naturally leads to variation in the pattern and path of the brachial plexus (Miller and Detwiler, 1936).

## Biomechanical basis of injury

Finite-element modelling has provided insight into how mechanical loads are transmitted to the neonatal plexus. Wright and Grimm (2024) demonstrated that small differences in anatomy can change how strain is distributed along the upper trunk under identical external loads. Their work showed that anatomical constraints can increase local deformation during traction. Iaconianni et al. (2024), using computer simulations of shoulder dystocia manoeuvres, demonstrated that lateral flexion, rotation and traction produce patterns of strain consistent with clinical BPBI. These models support the observation that traction and compression are sufficient to injure the plexus, producing the spectrum from neurapraxia to avulsion.

Clinical findings reinforce these biomechanical data. Pondaag et al. (2011) observed a strong association between birthweight and injury severity, suggesting that shoulder–pelvis fit and the complexity of extraction manoeuvres influence force transmission. However, high birthweight or dystocia does not always lead to injury. This suggests that another factor – neurologic susceptibility – may be necessary to fully explain the mechanical threshold for injury.

## Developmental anatomy as a predisposing factor

### Cervical ribs and first rib anomalies

A prospective study of 1138 women referred for an advanced ultrasound examination at a mean gestational age of 20.6 weeks found echogenic structures at the level of the seventh cervical vertebrae in 65% of foetuses, which were interpreted as rudimentary cervical ribs (Schut et al., 2026). Corroborating this high prenatal incidence, Bots et al. (2011) identified cervical ribs in around 40% of electively aborted foetuses, even in the absence of other abnormalities. It has been suggested that these cervical ribs may disappear by fusing with the vertebral body during postnatal growth (Chernoff and Rogers, 2004). However, Becker et al. (2002) identified complete cervical ribs in infants with BPBI at a greater proportion than the general population, and argued that these narrow the supracostoclavicular interval, predisposing adjacent nerves to traction injury. More than a third of the children with BPBI studied had a complete cervical rib (Becker et al., 2002). By modifying the anatomy of the region around the brachial plexus, a cervical rib may alter the direction of the vector of the stretching force, increasing its alignment to the axis of the most vulnerable brachial plexus bundle (Alfonso, 2011). Desurkar et al. (2011) reported congenital lower plexus palsy in neonates with non-ossified cervical ribs, the only identifiable abnormality.

These findings suggest that cervical ribs have a high prevalence in neonates, and both ossified and cartilaginous variants can distort plexus routing and predispose to injury. Population-level data reinforce the biological plausibility: cervical ribs occur in around 1% of the general population but in nearly 30% of patients with thoracic outlet syndrome (Masślanka et al., 2023). Exostoses of the first rib have also been reported to cause compression of the brachial plexus in neonates, resulting in BPBI, although this has only been described once (de Turckheim et al., 1991).

### Variant branching and scalene anomalies

Cadaveric studies in foetuses and infants demonstrate substantial variation in plexus branching, root divergence and fascicular relationships (Kirik et al., 2018; Uysal et al., 2003). These influence the curvature and anchoring points of the plexus, creating regions where traction forces may be concentrated. Connolly and Auchincloss (2021) outlined the embryology of first rib and scalene development, showing how anomalous insertions and fibrous bands contribute to corridor narrowing. Tense and hypertrophic scalene muscles have been identified as likely contributing factors in nTOS in the paediatric population (Rehemutula et al., 2015). These anomalies closely mirror the intraoperative findings in nTOS in adults, and similar patterns are increasingly recognized during neonatal BPBI explorations (Al-Hashel et al., 2013; Becker et al., 2002).

### Neurologic vulnerability

The finite element studies discussed earlier focused on angles of traction. However, narrowing around the plexus or tethering of the plexus owing to abnormal anatomy are likely to produce similar responses. There are no experimental data to date on this. A dual-factor model may explain the observed clinical variability: an infant with significant anatomic neurologic vulnerability exposed to dystocia forces may sustain BPBI. Another infant with the same developmental variant but an uneventful birth may remain asymptomatic. In some individuals this vulnerability may remain asymptomatic, only manifesting clinically following growth-related anatomical change or a secondary mechanical or inflammatory insult, resulting in symptoms subsequently labelled as nTOS. We propose that neonatal BPBI and adolescent nTOS represent distinct phenotypic expressions of a shared underlying anatomical vulnerability.

Not all BPBI cases result from neurogenic vulnerability. However, infants with these anatomical risk factors probably require significantly less strain to sustain an injury compared with those with normal anatomy. This is evident in those who develop nTOS in later life, where very few present in the absence of predisposing anatomy (Redman and Robbs, 2015).

### Developmental pathogenesis and genetic susceptibility

Developmental biology supports the concept that congenital narrowing of the thoracic outlet, such as cervical ribs, elongated C7 transverse processes, aberrant costoclavicular morphology or scalene–band anomalies, is not a random occurrence but a patterned developmental variant with a definable molecular basis. Vertebral and rib identity in the spine is regulated by *Hox* genes, which control somite segmentation and the capacity of a vertebra to form a rib (Burke et al., 1995; Wellik, 2009). *Hox* genes are a family of transcription factors which were first described in fruit flies for their ability to cause segmental homeotic transformations of the body plan (Lewis, 1963). Changes in the expression of these genes can produce predictable alterations in experimental models, including complete or partial rib formation on cervical vertebrae and morphological changes of the first thoracic rib (Burke et al., 1995; Rancourt et al., 1995; Wellik, 2009). Loss of function in *HoxB*-5 and dysregulated expression of *HoxB*-6 induce cervical rib formation in mice, disrupting the normal C7–T1 boundary (Rancourt et al., 1995). Broader disruption of the *Hox* code have been shown to remodel axial patterning across multiple vertebral levels (Burke et al., 1995). These findings provide a biologically coherent explanation for the familial clustering of cervical rib and first-rib anomalies that clinicians increasingly observe in BPBI and nTOS populations (Goeteyn et al., 2021).

Although specific pathogenic variants are rarely identified clinically, radiographic population studies consistently demonstrate heritability in cervical rib prevalence, consistent with polygenic modulation of these developmental pathways rather than single-gene causation (Galis et al., 2025; Partiot et al., 2020). Patients with TOS and cervical ribs have also been found to have more miscarriages than those without, which may be explained by disturbance of the expression of multiple genes, including *Hox* genes (Schut et al., 2020).

Taken together, these developmental and molecular findings reinforce the clinical observation that congenital neurogenic vulnerability factors arise from patterned embryological processes rather than incidental variation. They provide a plausible biological process for familial clustering and strengthen the concept of a congenital ‘susceptibility phenotype’ present before birth. This framework aligns with the broader thesis of this review: that BPBI arises not solely from obstetric mechanics but from the interaction between delivery forces and a pre-existing anatomical predisposition that is biologically determined during axial patterning in early embryogenesis. Identification of this predisposition may allow for extra precautions to be taken during deliveries to decrease the incidence of BPBI (Tzou et al., 2014).

## Clinical implications

### Diagnostic challenges and clinical assessment

Brachial plexus birth injury frequently results from complex biomechanical forces during delivery, many of which are beyond the delivering clinician’s control (Chater et al., 2004). Consequently, understanding the contribution of anatomical predisposition is likely to become a key component of prenatal assessment. However, familiar findings such as supraclavicular fullness, focal tenderness or Tinel’s sign over the scalene interval are not feasible in this population. While targeted imaging of the brachial plexus is not standard assessment in any clinical setting, Tzou et al. (2014) report that cervical ribs can be identified during the prenatal screening sonography. Cai et al. (2022) demonstrated that magnetic resonance imaging (MRI) can also be used to diagnose foetal vertebral anomalies with improved accuracy over ultrasound, although no cases of cervical rib were reported. Regarding the brachial plexus itself, MRI may demonstrate supraclavicular crowding or plexus tenting, but these signals are often lost in the T2 high-signal assessment. The cervical spine at birth is often not ossified sufficiently for it to be possible to identify cervical ribs or elongated transverse processes on radiographs (Lustrin et al., 2003). Identification of these anatomical abnormalities with focussed screening in pregnancies with known risk factors (macrosomia, previous shoulder dystocia or maternal gestational diabetes) could further assist in informing the risk of BPBI and shoulder dystocia in attempted vaginal delivery (Crofts et al., 2012). If a genetic marker for these anatomical variations were identified, existing prenatal tools (such as expanded carrier screening, cell-free DNA, chromosomal microarray analysis and next-generation sequencing) could be used to guide decision-making for mothers and obstetricians (Makhamreh et al., 2025).

### Surgical considerations and management strategies

In the select cohorts which undergo neonatal brachial plexus exploration, surgeons often do not actively seek or recognize these characteristics of anatomic variation in the traumatized surgical field (Desurkar et al., 2011; Rehemutula et al., 2015; Tzou et al., 2014). The primary aim of the surgeon is to identify the level and severity of the nerve injury within a scarred surgical field lacking normal anatomy (Bahm et al., 2007). However, it is clinically plausible that a percentage of these cases involve anatomical findings analogous to adult nTOS, which may serve as a significant contributory factor. In such cases, it could be argued that decompression and neurolysis may be beneficial in the place of standard neuroma resection or nerve grafting. While prospective data are lacking, surgical teams report improved nerve excursion and reduced secondary tethering when anatomical compression is addressed at the time of reconstruction. This approach is biologically logical: a recovering nerve swollen within a narrow corridor is vulnerable to chronic entrapment physiology (Mackinnon, 2002).

## Conclusion

We propose that developmental anatomical variation may contribute to neurologic vulnerability in the aetiology of BPBI. This concept offers a potential explanation for why some infants with recognized obstetric risk factors sustain injury while others do not, and may account for the range of severity observed in some neonates.

Consequently, this topic merits discussion within the hand surgery and obstetric communities. Introducing anatomical assessment into neonatal examination should be considered. Further research is needed to explore whether anatomical variation does in fact confer neurologic vulnerability. Future studies might investigate whether biological markers during pregnancy signal such a vulnerability to aid obstetric decision-making. Additionally, exploring whether clinical markers in parents, such as signs of nTOS or anatomical variation, correlate with foetal risk may offer the potential to enhance prenatal diagnostic accuracy, refine operative management and improve long-term outcomes.
